# Valorization of Mussel Shell Waste to Chitin, Chitosan, and Calcium Lactate for Bio-Green-Circular Management

**DOI:** 10.3390/ijms27083627

**Published:** 2026-04-18

**Authors:** Chaowared Seangarun, Somkiat Seesanong, Banjong Boonchom, Wimonmat Boonmee, Sirichet Punthipayanon, Nongnuch Laohavisuti, Pesak Rungrojchaipon

**Affiliations:** 1 Material Science for Environmental Sustainability Research Unit, School of Science, King Mongkut’s Institute of Technology Ladkrabang, Bangkok 10520, Thailand; chaowared@gmail.com (C.S.); sirichet@g.swu.ac.th (S.P.); pesak.ru@kmitl.ac.th (P.R.); 2 Office of Administrative Interdisciplinary Program on Agricultural Technology, School of Agricultural Technology, King Mongkut’s Institute of Technology Ladkrabang, Bangkok 10520, Thailand; somkiat.se@kmitl.ac.th; 3 Municipal Waste and Wastewater Management Learning Center, School of Science, King Mongkut’s Institute of Technology Ladkrabang, Bangkok 10520, Thailand; 4 Department of Chemistry, School of Science, King Mongkut’s Institute of Technology Ladkrabang, Bangkok 10520, Thailand; 5 Department of Biology, School of Science, King Mongkut’s Institute of Technology Ladkrabang, Bangkok 10520, Thailand; wimonmat.bo@kmitl.ac.th; 6 Department of Sports Science, Faculty of Physical Education, Srinakharinwirot University, Bangkok 10110, Thailand; 7 Thailand Association of Mixed Martial Arts, 431/19 Rajchadapisek Road, Yannawa, Chongnonsee, Bangkok 10120, Thailand

**Keywords:** mussel shell waste, chitin, chitosan, calcium lactate, upcycling, sustainable waste management

## Abstract

This study presents a green bio-upcycling strategy for converting mussel shell biowaste into three value-added products: chitin, chitosan, and calcium lactate. Mussel shells were treated chemically with lactic acid during demineralization, yielding a solid fraction rich in chitin and a liquid fraction containing calcium and lactate ions. The solid fraction was sequentially purified by deproteinization and decolorization, then deacetylated to obtain chitosan, while the liquid fraction was evaporated to obtain calcium lactate. Notably, 2.37 g of raw chitin, 2.15 g of purified chitin, and 275.87 g of calcium lactate were obtained from 100 g of mussel shells, demonstrating the efficiency of the process. FTIR spectra revealed characteristic absorption bands corresponding to α-chitin and chitosan functional groups, while XRD patterns indicated the crystalline α-chitin structure and the formation of calcium lactate pentahydrate. TGA demonstrated the high thermal stability of chitin and chitosan and confirmed the presence of crystallization water in calcium lactate. In conclusion, these results confirmed the successful preparation of α-chitin, chitosan, and calcium lactate pentahydrate, with improved purity compared to previous studies. This approach highlights the potential of the green bio-upcycling process of mussel shell waste as a renewable source for the eco-friendly production of biopolymers and calcium salts, supporting sustainable waste management and the development of the Bio-Circular-Green (BCG) economy.

## 1. Introduction

The application of green chemistry in biorefining processes is expanding worldwide [1]. Green chemistry emphasizes minimizing environmental harm and the safety and well-being of all living organisms [2]. Food waste accumulates in large quantities every day and poses an escalating challenge for waste management systems and environmental health. One of the enormous amounts of food waste, bivalve shells, occur every day and are interesting topics for recycling and upcycling to advance a resource-efficient, zero-waste, and carbon-neutral circular economy, while also fostering sustainable investment opportunities [3]. In particular, mussels (*Perna viridis*), a type of bivalve mollusk, are widely consumed as seafood in Thailand. The Thai framer produces more than approximately 50,000 tons of mussels annually from aquaculture farms [4]. However, only about 30% of the total weight is the edible portion, while the remaining 70% consists of non-edible shells, which are typically discarded as biowaste. Consequently, it is estimated that around 35,000 tons of shell waste is generated each year in Thailand, posing environmental and waste management challenges, but this situation also offers opportunities for sustainable recycling and upcycling [5,6]. The uncontrolled disposal of discarded shells causes various environmental and health problems, such as odor emission, contamination from organic decay, and undesirable visual impacts [7]. Moreover, the progressive microbial degradation of organic matter ultimately produces several greenhouse gases, namely CO_2_, NO_x_, NH_3_, and H_2_S [8]. Several studies reported that the primary content of mussel shell is CaCO_3_, leading to the development of various routes for converting mussel shell waste into value-added calcium products, such as CaO [9,10,11], highly pure CaCO_3_ [12], calcium phosphates [13], calcium acetate [14], calcium lactate [15], calcium citrate [16], and calcium sulfate [17], which can be used in many applications [18,19]. However, most of these approaches focus on single-product recovery and often overlook the co-existing biopolymer fraction (e.g., chitin), resulting in inefficient and incomplete resource utilization [20,21].

Chitin, the second-most abundant natural polysaccharide on Earth, consists of repeating β(1,4)-N acetylglucosamine units, which have been increasingly popular as an alternative to nonrenewable materials [22,23,24]. Chitin has high hydrophobicity due to its crystalline structure, resulting in its insolubility in water, organic, and inorganic solvents [25,26]. The limited solubility of chitin negatively affects the production of chitin-based products or applications for various industries [27]. Consequently, chitin was converted into chitosan, a more soluble derivative that has biodegradability, biocompatibility, non-toxicity, adsorption, antioxidant, humectant, and antimicrobial activity [28]. In general, the commercial method for chitosan extraction from raw materials (crab and shrimp shells) was achieved by a multiple-step chemical process, including: demineralization by strong acids, a deproteinization process through alkaline solutions, a decolorization process through bleaching agents, and a deacetylation process through very strong alkaline solutions to convert chitin into chitosan. Generally, after demineralization, calcium carbonate (CaCO_3_) from natural sources dissolves as Ca^2+^ ions in aqueous solution, while chitin remains in its solid form [29]. So far, few studies have focused on the Ca^2+^ ion that remains in wastewater. Recently, mussel shells were used as raw material to extract chitin and chitosan, and a large amount of Ca^2+^ ions were released during this process [15,30,31]. Consequently, Ca^2+^ ions were used to prepare calcium acetate, demonstrating the feasibility of producing multiple value-added compounds from a single biowaste source [32]. Building on this achievement, the present study aims to further develop and expand the concept by producing calcium lactate, a higher-value and more widely used calcium compound than calcium acetate, together with chitin and chitosan in a single integrated process, which indicates a sample model of a circular economic process for industrial-scale production to reduce waste and use resources more efficiently [32].

Lactic acid (CH_3_CHOHCOOH) is one of the most widely used organic acids across various industries due to its versatile applications [33], particularly showing potential as a demineralizing agent for extracting chitin from crustaceans such as shrimps, prawns, and crabs in previous research [34,35,36]. Moreover, Ca^2+^ and CH_3_CHOHCOO^−^ ion aqueous solution can be used to produce calcium salts of lactic acid, which is called “calcium lactate”, reported by McReynolds et al. [36]. They have successfully extracted chitin from crab shells and converted the calcium solution from the demineralization process into calcium lactate via precipitation. Calcium lactate possesses superior characteristics compared to many other calcium salts, particularly its high solubility, excellent bioavailability, and low toxicity [15]. These properties enable its versatile applications, ranging from use as a food additive, stabilizer, thickening agent, and dietary supplement [37], to a calcium source for animals and plants [38]. In addition, calcium lactate has been employed in water treatment processes as a precipitating agent for suspended solids [39], construction materials to enhance compressive strength and decrease water permeability of concrete [40], and raw material to synthesize advanced compounds such as calcium-containing polymer compounds [41]. Due to the wide-ranging benefits and industrial significance of calcium lactate, it has attracted considerable attention for its high demand and economic value [42]. Consequently, lactic acid was selected as a demineralizing agent to simultaneously extract chitin/chitosan from mussel shells and generate calcium lactate, which is reported in this work for the first time. This study highlights the green bio-upcycling of mussel shells as a renewable precursor for the simultaneous production of chitin, chitosan, and calcium lactate, underscoring the potential of biowaste as a key resource for sustainable waste management and the production of value-added materials. Lactic acid was employed as a green demineralizing agent for mussel shells, yielding a suspension comprising a solid chitin-rich fraction and a liquid phase enriched in calcium and lactate ions. These two fractions were subsequently separated by simple filtration. The solid fraction undergoes sequential purification and deacetylation to produce chitosan, while the liquid fraction is recovered through evaporation to obtain high-purity calcium lactate crystals. The products obtained, namely chitin, chitosan, and calcium lactate, were characterized using Fourier Transform Infrared Spectroscopy (FTIR), X-Ray Diffraction (XRD), Thermogravimetric Analysis (TGA), and Scanning Electron Microscopy (SEM), to confirm their chemical and physical properties. The results will be compared with chitin, chitosan, and calcium lactate obtained from other methods and other precursors in previous research, such as shrimp shells [43,44], crab shells [36], and other shell sources [45]. It is hypothesized that mussel shell waste can be efficiently transformed into chitin, chitosan, and calcium lactate through a single integrated process using mussel shell as an alternative biopolymer and calcium source, thereby enhancing overall resource utilization and reducing waste generation compared to commercial methods. This study not only takes a further step toward developing sustainable waste management that aligns with the Bio-Circular-Green economy (BCG) but also presents an alternative chitin, chitosan, and calcium source for use across various industries.

## 2. Results and Discussion

### 2.1. Chitin and Chitosan Production Results

**Figure 1:**
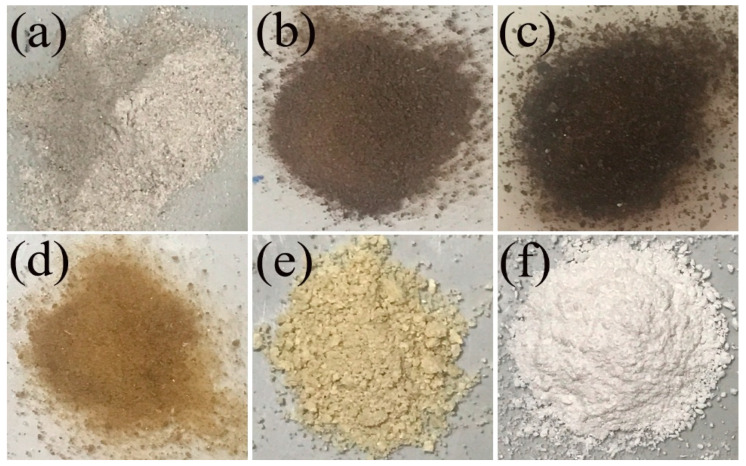
Images of mussel shell powders (MSP): (**a**) raw chitin (RCH), (**b**) deproteinized chitin (DPC), (**c**) purified chitin (PCH), (**d**) chitosan (CTS), (**e**) calcium lactate (CCL), and (**f**) derived from mussel shells.

Figure 1 shows the colors of MSP (a), RCH (b), DPC (c), PCH (d), CTS (e), and CCL (f) powders, which appeared as white-gray, dark brown, dark black, pale brown, pale yellow, and white, respectively. The extraction of 100 g of MSP was found to be 2.37 (RCH), 2.30 (DPC), 2.15 (PCH), and 2.10 (CTS) g. The decreasing amount results from the removal of impurities in each extraction process of RCH to CTS forms. Moreover, the apparent color of chitin and chitosan changes from dark brown to pale yellow after each process, as shown in Figure 1, confirming the removal of proteins, pigments, and impurities, as well as deacetylation. The apparent colors of chitin and chitosan obtained in this work are different from those extracted from mussel shells by using demineralization with acetic acid, as reported by Seangarun et al. [32]. Additionally, the amounts of chitin/chitosan obtained in this work are lower than that reported by Seangarun et al. [32] (3.02/2.70%) and obtained from shrimp shells (7–14%).

The CTS sample exhibited a degree of deacetylation (DD) of 88%, reflecting a highly deacetylated structure, which is higher than that reported by Seangarun et al. (86%) [32]. In general, chitosan with a DD exceeding 85% is considered advantageous for applications that demand enhanced solubility and cationic functionality, including biomedical materials, drug carriers, and antimicrobial films [45,46]. This DD value in this work is consistent with those previously reported by Triunfo et al. (83–93%) [45], but noticeably higher than that obtained by Kaewprachu and Jaisan (32–52%) [30]. The Mw of the CTS sample was estimated at approximately 85 kDa, which is higher than the value reported by Seangarun et al. (72 kDa) [32]. From the results of chitin and chitosan production from mussel shells, the percentage yield, apparent colors, degree of deacetylation (DD), and molecular weight (Mw) obtained in this work are different from our previous report, suggesting they have been affected by different acids in the demineralization process. The FTIR, XRD, TGA, and SEM results will be presented in the following sections to confirm the chemical and physical properties of the obtained products.

### 2.2. Calcium Lactate Production Results

The weight of CCL is 275.87 g obtained from the evaporation of 2000 mL of the liquid fraction from the demineralization process. Based on the experiment data, the percentage yield of CCL production was estimated according to Equation (1) [31] by using calcium ions (0.96 M) as the determining limiting reagent for the formation of calcium lactate pentahydrate, which was found to be 94.31%.

Ca^2+^(aq) + 2CH_3_CHOHCOO^−^(aq) + 5H_2_O(l) → Ca(CH_3_CHOHCOO)_2_·5H_2_O(s) (1)

The inorganic elemental composition of the prepared calcium lactate (CCL) was identified by the X-ray fluorescence (XRF) technique. The XRF result shows that CCL mainly contains 98.3% CaO. In addition, 1.7% of other minerals were found, including 0.594% Na_2_O, 0.062% MgO, 0.019% Al_2_O_3_, 0.044% SiO_2_, 0.077% P_2_O_5_, 0.190% SO_3_, 0.035% Cl, and 0.696% SrO. Compared to the previous work, the calcium oxide (CaO) content of mussel shell-derived calcium lactate analyzed by XRF in this work (98.3%) was higher than that of the previous work (94–96%), while other mineral contents decreased [31]. It should be noted that previous studies prepared calcium lactate directly from mussel shells without concurrently extracting chitin or chitosan, whereas the present work successfully produced all three value-added compounds in a single integrated process. The calcium compound synthesis process developed in this study demonstrates superior potential compared to previous research due to the higher calcium content of the resulting product. This enhanced calcium content not only shows the effectiveness of the synthesis method but also makes the compounds more suitable for downstream applications, particularly in industries that demand higher quality standards, such as pharmaceuticals, biomedical materials, and food additives [47,48].

### 2.3. The Fourier Transform Infrared (FTIR) Results

**Figure 2:**
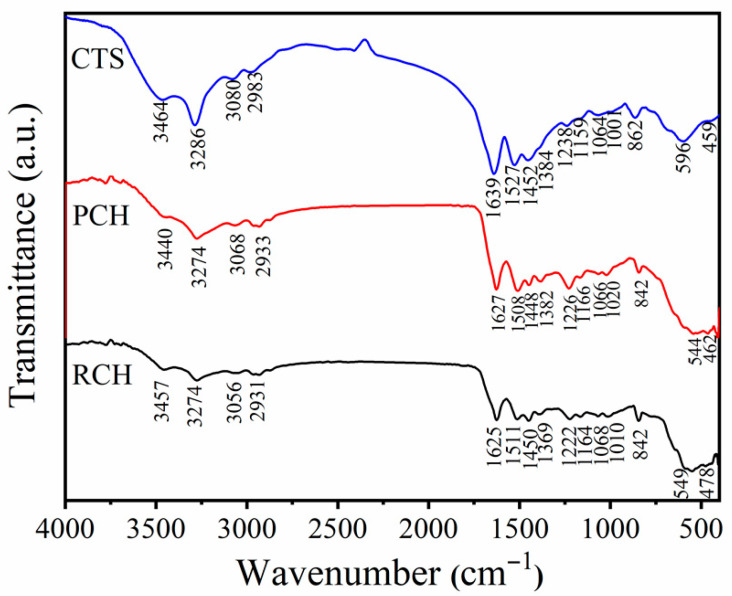
FTIR spectra of raw chitin (RCH), purified chitin (PCH), and chitosan (CTS) samples extracted from mussel shells.

A Fourier Transform Infrared Spectrometer (FTIR) was used to investigate the vibrational characteristics of raw chitin (RCH), purified chitin (PCH), and chitosan (CTS) extracted from mussel shells by lactic acid demineralization, which are slightly different from those demineralized by acetic acid, as reported in our previous work [32]. The spectra resulting from all prepared samples are shown in Figure 2. For raw chitin (RCH), three characteristic peaks of amide groups were found, confirming the functional groups of chitin [49]. The characteristic peak of amide I, which is found at 1625 cm^−1^, could be attributed to C=O stretching vibrations. The peak of amide II was found at 1511 cm^−1^, attributed to N-H bending. The peak of amide III was found at 1369 cm^−1^, attributed to C-N stretching [50]. The broad peak at around 3457 cm^−1^ is attributed to O-H stretching [51]. The vibration of N-H stretching was split into two peaks, which can be observed at 3274 and 3056 cm^−1^. These peaks are similar to the extracted α-chitin by Focher et al. [50], where the splitting of peaks can be clearly visible in the spectrum of α-chitin, but are weak and not easily found in β-chitin. Additionally, other vibrational peaks of chitin molecules were also observed. The observed peaks at 2931, 1450, 1164, 1010, and 842 cm^−1^ are assigned as C-H stretching, H-C-H bending, asymmetric stretching of C-O-C bridge, C-O stretching, and H-C-H out-of-plane bending, respectively [52,53]. The characteristic peaks of purified chitin (PCH) are similar to those of raw chitin (RCH) with slightly different positions, confirming the similarity of functional groups after being treated with chemicals to eliminate protein and pigment. The FT-IR bands observed are closely matched to previous reports on chitin and chitosan from crustaceans and insects [43,49].

After the deacetylation of chitin from mussel shells using NaOH, the FTIR spectra of chitosan (CTS) show similar bands, but certain differences could be found in the wavelength and adsorption intensity [49]. Similar to chitin, the characteristic peaks of amide I, amide II, and amide III are observed at 1639, 1527, and 1384 cm^−1^. The observed peak of O-H stretching was found at 3457 cm^−1^, while the peaks of N-H stretching are observed at 3286 and 3080 cm^−1^. The peaks of C-H stretching, H-C-H bending, asymmetric stretching of C-O-C bridge, C-O stretching, and H-C-H out-of-plane bending are also observed at 2983, 1452, 1159, 1001, and 862 cm^−1^, respectively.

**Figure 3:**
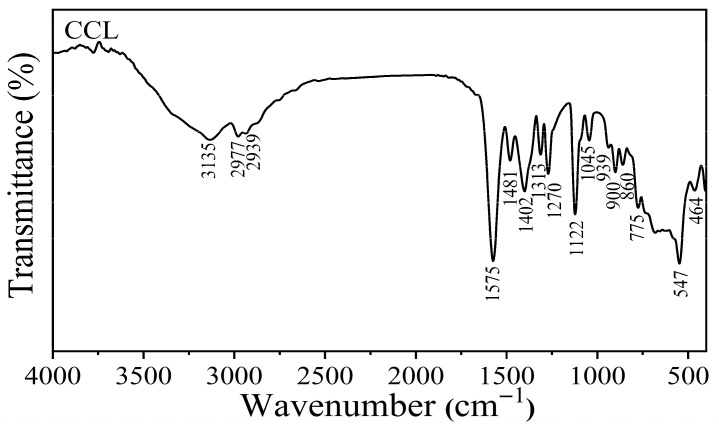
FTIR spectrum of calcium lactate (CCL) from the demineralization process of the extraction of mussel shells.

The spectrum of calcium lactate (CCL) contains the functional groups of CH_3_CHOHCOO^−^, H_2_O and Ca-O, which is shown in Figure 3 [31]. All vibrational peaks were then explained. The broad peak at 3135 cm^−1^ was assigned to the O-H stretching modes of H_2_O. The weak peaks at 2977 and 2939 cm^−1^ were assigned to the asymmetric and symmetric stretching vibrations of the C-H bond. The two sharp peaks observed at 1575 and 1481 cm^−1^ were attributed to the asymmetric and symmetric stretching modes of the carboxylate group (large dipole moment). The peak at 1122 cm^−1^ was assigned to the C-O stretching vibrations. These results are similar to calcium lactate derived from mussel shells reported by Mititelu et al. [15]. The peaks at 1313 and 1270 cm^−1^ were assigned as C-H bending. The weak peaks between 1060 and 850 cm^−1^ were assigned as C-C stretching of C-CH_3_ and C-COO^−^ groups [31]. The broad adsorption band between 500 and 900 cm^−1^ is similar to the extracted calcium lactate from shrimp shells by Dechapinan et al. [54], which may include the stretching of metal oxide groups (Ca-O stretching). In summary, all vibrational characteristic peaks are highly similar to those of calcium lactate from mussel shells derived by other methods in previous research [15,31].

### 2.4. X-Ray Diffraction (XRD) Results

**Figure 4:**
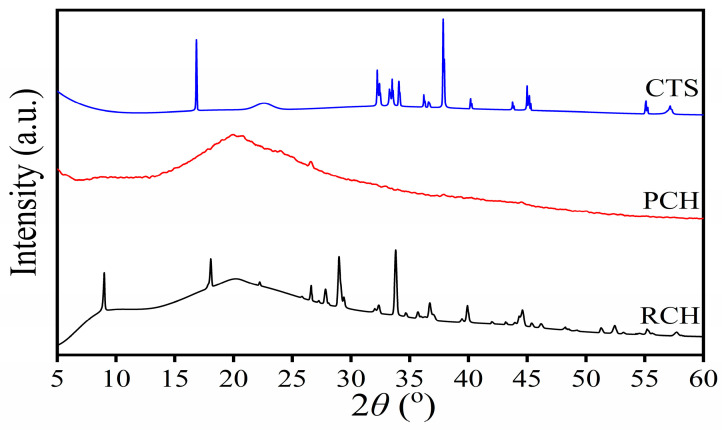
XRD patterns of raw chitin (RCH), purified chitin (PCH), and chitosan (CTS) samples extracted from mussel shells.

The XRD patterns of the prepared raw chitin (RCH), purified chitin (PCH), and chitosan (CTS) in this work are shown in Figure 4, which are slightly different from those obtained from mussel shell extracted by acetic acid in the mineralization process, reported by our previous work [32]. No significant differences were found in the spectra between unbleached and bleached chitin. The XRD patterns of RCH and PCH exhibit broad diffraction peaks at 2θ = 9.0° and 20.2°, which distinguish crystalline peaks overlapped with an amorphous domain. These diffraction patterns were in agreement with those reported for chitin extracted from other natural sources in previous works, corresponding to the (020) and (110) crystalline planes, respectively. Yen and Mau [55] extracted chitin from crab shells and found two broad peaks at 2θ = 9.3° and 19.1°. Likewise, Triunfo et al. [45] extracted chitin from larvae, pupal exuviae, and adult insects (Hermetia illucens) and also observed similar broad peaks at around 2θ = 9° and 19°. These results show that the XRD pattern of chitin extracted from various natural sources exhibits two similar broad peaks at 2θ values of about 9–10° and 19–20°. After deacetylation, the XRD pattern of CTS shows sharp peaks at 18°, 38°, and 44°, corresponding to those of commercial chitosan reported by Suneeta et al. [44]. In addition, a weak broad peak was found at 23^o^, which is different from the chitosan extracted in previous research [44,45].

**Figure 5:**
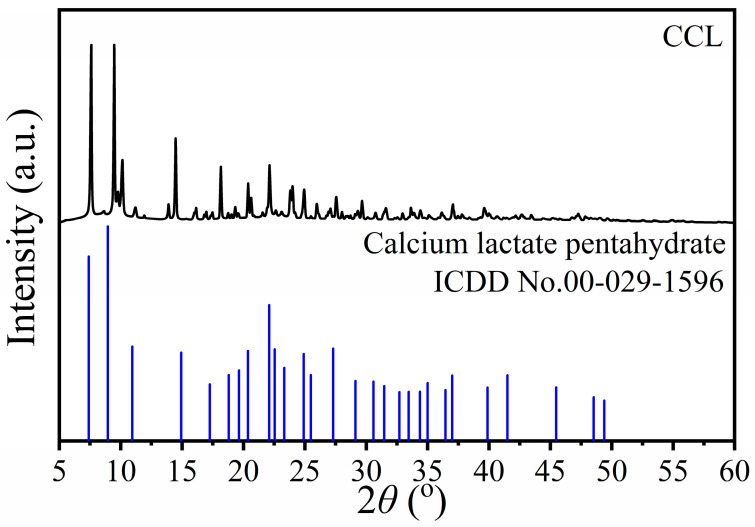
XRD pattern of calcium lactate (CCL) from the demineralization process of the extraction of mussel shells.

XRD was used to investigate the crystallography of CCL produced by using calcium solution from the demineralization process, as shown in Figure 5. The resulting diffractograms were then employed to verify the chemical structure by comparing it with the international diffraction databases. As a result, the diffraction pattern is in agreement with the existing ICDD card no. 00-029-1596, which was assigned to calcium lactate pentahydrate (Ca(CH_3_CHOHCOO)_2_·5H_2_O) [56,57]. In addition, some different diffraction peaks, such as at 2θ of 11.30 and 14.11°, were observed with low intensity. The different peaks of calcium lactate observed in this work are due to the different enantiomeric forms of calcium lactate, which were also found in previous works [31,56]. According to Tansman et al., there are two different diffraction patterns named “calcium L-lactate pentahydrate and calcium D-lactate pentahydrate” depending on the enantiomeric characteristic of lactate (CH_3_CHOHCOO^−^) anions. In addition, mixed-enantiomeric forms of Ca(CH_3_CHOHCOO)_2_·5H_2_O between D- and L-CH_3_CHOHCOO^−^ were also observed in the form of peaks that were different from the standard ICDD card no.00-029-1596, which was called calcium DL-lactate pentahydrate [31,56]. Therefore, the diffraction pattern of the prepared calcium lactate in this work provided the Ca(CH_3_CHOHCOO)_2_·5H_2_O with both DL- and L- enantiomeric forms. This result is similar to the diffraction patterns of calcium lactate prepared by Seesanong et al. [31], using the reaction between mussel shell wastes and various concentrated lactic acids (6, 8, and 10 M). These results strongly support that the demineralization solution (DMS) from the demineralization process of mussel shells was successfully used to prepare calcium lactate pentahydrate (Ca(CH_3_CHOHCOO)_2_·5H_2_O).

### 2.5. Thermogravimetric Analysis (TGA) Results

**Figure 6:**
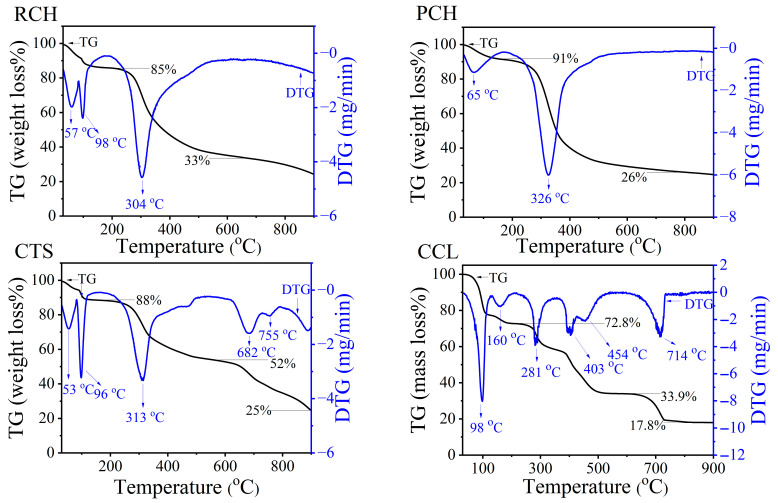
TG/DTG curves of raw chitin (RCH), purified chitin (PCH), chitosan (CTS), and calcium lactate (CCL) samples extracted from mussel shells.

Thermogravimetric Analysis (TGA) is an important method to determine thermal stability, the nature of the degradation process, the effect of polymer sources, and the effect of additives on degradation temperature. Thermal decomposition (TG/DTG) traits of raw chitin (RCH), purified chitin (PCH), and chitosan (CTS) from mussel shells extracted by lactic acid in the mineralization process for this work in the temperature range of 30–900 °C are shown in Figure 6, which shows the difference in those obtained from mussel shells extracted by acetic acid in the demineralization process, reported in our previous work [32]. For RCH, the first thermal decomposition step was observed in the temperature range of 30–163 °C with DTG peaks at 57 and 98 °C, 14% mass loss, which corresponds to the evaporation of water (H_2_O) in the chitin structure [57]. Then, the second thermal decomposition step was observed in the temperature range of 180–514 °C with a DTG peak at 304 °C and 61% mass loss, which corresponds to different degradation reactions of chitin, including polysaccharide depolymerization, thermal cracking, ring opening, and the deterioration of acetylated chitin units [58]. PCH also showed thermal decomposition in the range between 30 and 161 and 180–510 °C with 10% and 65% mass loss, respectively. The first and second thermal decomposition steps are due to the evaporation of water in the chitin structure and polymer degradation, respectively [57,58]. The thermal decomposition of PCH shows a similar pattern compared to that of RCH, with slight variations after purification by deproteinization and bleaching processes. In comparison, the degradation of chitin from mussel shells in this work was lower than the previous studies, which have previously been reported in the range of 350–390 °C, depending on their biological origin, from 350 °C in shrimp shells [59] to 372 °C in crab shells [60], and 390 °C in grasshoppers [61].

For the thermal decomposition of CTS, the first thermal decomposition step was observed in the temperature range of 30–106 °C with DTG peaks at 53 and 96 °C and 12% mass loss, which corresponds to the elimination of water (H_2_O) in the chitosan structure [57]. The second thermal decomposition step was observed in the temperature range of 190–502 °C with a DTG peak at 313 °C and 63% mass loss, which corresponds to the degradation of amino groups forming volatile compounds such as ammonia (NH_3_) [62] and the degradation of the polymer structure, including polysaccharide depolymerization, thermal cracking, and ring opening [58]. In this step, the mass loss of chitosan is lower than that of chitin because the acetyl groups (-COCH_3_) are already eliminated after the deacetylation process to convert chitin into chitosan [62]. At the third decomposition stage in the temperature range of 520–900 °C with DTG peaks at 682 and 755 °C, the observed weight loss of about 27% was attributed to the carbonization of residual organic matter, producing volatile species such as acetic acid, butyric acid, and short-chain fatty acids (mainly C_2_–C_6_) [62,63], while the remaining residue (approximately 18%) corresponded primarily to thermally stable inorganic compounds composed of carbon, nitrogen, and oxygen [64]. In this study, the final residue of CTS was approximately 25%, which is noticeably lower than those reported in previous research [32]. For instance, Barbosa et al. [62] observed a final residue of 30–33% for chitosan samples derived from shrimp shells.

**Figure 7:**
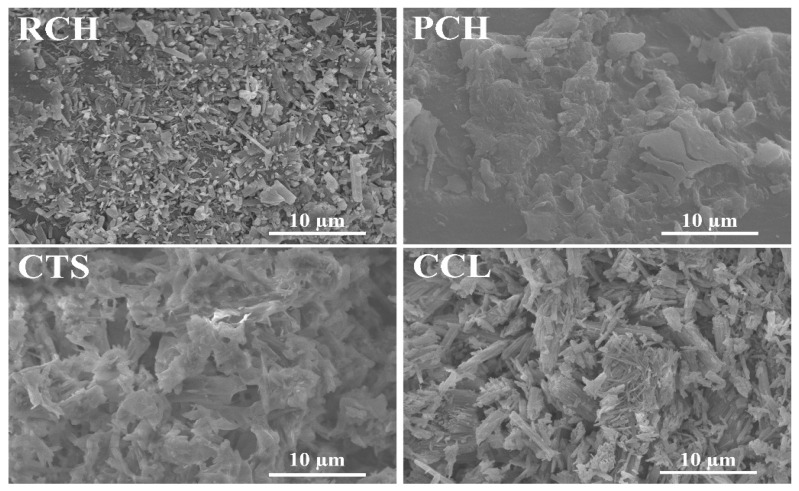
SEM images of raw chitin (RCH), purified chitin (PCH), chitosan (CTS), and calcium lactate (CCL) samples extracted from mussel shells.

Thermal decomposition (TG/DTG) of CCL extracted from mussel shells in the temperature range of 30–900 °C are shown in Figure 7. The first thermal decomposition step was observed in the temperature range of 30–200°C with DTG peaks at 98 and 160 °C and 27.2% mass loss, which corresponds to the elimination of the hydrated water (5H_2_O) that existed in the crystal structure of calcium lactate pentahydrate (Ca(CH_3_CHOHCOO)_2_·5H_2_O) with 29.22% of theoretical mass loss [65,66]. The second thermal decomposition step occurred in the temperature range of 210–550 °C with DTG peaks at 281, 403, and 454 °C and 38.9% mass loss, which corresponds to the elimination of CH_3_CHOHCOOC_2_H_5_ and the formation of CaCO_3_ during the complex thermal decomposition of anhydrous calcium lactate (Ca(CH_3_CHOHCOO)_2_), which presents many DTG peaks [67,68]. The third thermal decomposition step was observed in the temperature range of 580–750 °C with a DTG peak at 714 °C and 16.1% mass loss, which corresponds to the “decarbonation process” of calcium carbonate (CaCO_3_), resulting in the elimination of CO_2_ gases and formation of calcium oxide (CaO) [69]. The mechanisms of these thermal decomposition steps are present in Equations (2)–(4):

Dehydration (30–200 °C):

Ca(CH_3_CHOHCOO)_2_⋅5H_2_O(s) → Ca(CH_3_CHOHCOO)_2_(s) + 5H_2_O(g)↑ (2)

Ethyl-lactate elimination (210−550 °C):

Ca(CH_3_CHOHCOO)_2_(s) → CaCO_3_(s) + CH_3_CHOHCOOC_2_H_5_(g)↑ (3)

Decarbonization (580−750 °C):

CaCO_3_(s) → CaO(s) + CO_2_(g)↑ (4)

The thermal decomposition tags of CCL are in line with those of calcium lactate from mussel shells derived by Seesanong et al. [31]. However, the total mass loss observed in this study (approximately 82%) was higher than that reported by Seesanong et al. (77–79%), suggesting that the CCL obtained here contained a higher proportion of crystalline hydrate and a lower content of thermally stable inorganic impurities. In addition, the TG data are in agreement with the XRD pattern of CCL, which exhibits the diffraction of crystalline calcium lactate pentahydrate (Ca(CH_3_CHOHCOO)_2_·5H_2_O) in the previous section. Therefore, it can be indicated that calcium lactate pentahydrate (Ca(CH_3_CHOHCOO)_2_·5H_2_O) was successfully produced from the demineralization solution (DMS) in the chitin extraction processes.

### 2.6. Scanning Electron Microscope (SEM) Results

The surface morphologies of the raw chitin (RCH), purified chitin (PCH), chitosan (CTS) and calcium lactate (CCL) from mussel shells were analyzed by Scanning Electron Microscopy (SEM) at 10,000× magnification and the SEM images are shown in Figure 7. The morphologies and shapes of all obtained products in this work are significantly different from those obtained from mussel shell extracted by acetic acid in the mineralization process, reported by our previous work [32]. The extracted RCH surface shows many small particles of various shapes and sizes. However, after purification (NaOH deproteinization and H_2_O_2_ decolorization), the surface of PCH is smooth, and many small particles on the chitin surface have disappeared. The morphology of PCH in this study is consistent with that of α-chitin extracted from shrimp and crab shells reported by Mohan et al. [70], where purified chitin exhibited well-defined fibrillar microstructures with reduced porosity and smoother surfaces after purification. The morphology of CTS shows the shapeless sheets of polymer in different sizes, which closely resemble the SEM morphology of chitosan derived from shrimp shells reported by de Queiroz et al. [71].

The morphologies of calcium lactate (CCL) exhibit timber-like microparticles with varying sizes and some irregular shapes, which may result from imperfections in the crystal formation during the crystallization process. The particle morphology of CCL is comparable to that of calcium lactate synthesized from mussel shells using concentrated lactic acid, as reported by Seesanong et al. [31]. However, the particle sizes obtained in this study were smaller than those described in the previous work.

## 3. Materials and Methods

### 3.1. Materials and Reagent Preparation

Mussel shells were collected from Ang Sila seafood market, Chonburi province, Thailand. The mussel shell wastes were soaked in water to remove dust and dirt. After that, the cleaned mussel shells were ground in a mortar, sieved through a 100-mesh sieve, and dried in an oven at 100 °C for 2 h. Finally, the obtained mussel shell powders (MSPs) were checked for purity by X-ray fluorescence (XRF, SRS 3400, Bruker, Billerica, MA, USA), which showed 96% CaO.

All chemicals used in this work are commercial-grade and not further purified. Lactic acid (CH_3_CHOHCOOH, 88% *w*/*w*, Sigma-Aldrich, Burlington, MA, USA) was diluted to 1 M with deionized water for use in the demineralization process. Sodium hydroxide (NaOH, 98%, Merck/Burlington, MA, USA) pellets were dissolved in deionized water to prepare a 10 M NaOH solution for deacetylation, then diluted to 1 M for deproteinization. Hydrogen peroxide (H_2_O_2_, 50% *w*/*v*, Merck/Burlington, MA, USA) was diluted with deionized water to obtain 10% *w*/*v* H_2_O_2_ solution for use in the decolorization process. Finally, ethanol (C_2_H_5_OH, 99%, Carlo Erba, Normandy, France) was used as a chitosan solidification agent.

### 3.2. Mussel Shell Extraction

Let us start with the first demineralization process: MSP containing 96% calcium carbonate (CaCO_3_) content was treated with lactic acid according to Equation (1). In the conventional way, 100 g of MSP was digested with 2000 mL of 1 M lactic acid by magnetic stirrer at 600 rpm for 2 h without temperature control. The reaction ends (Equation (5)) when no more carbon dioxide bubbles are formed, and two phases appear: a solid phase and a liquid phase. The liquid fraction contains a solution of calcium (Ca^2+^) and lactate (CH_3_CHOHCOO^−^) ions, while the remaining solid fraction mainly contains chitin.

CaCO_3_(s) + 2CH_3_CHOHCOOH(aq) → Ca(CH_3_CHOHCOO)_2_(aq) + H_2_O(l) + CO_2_(g)↑ (5)

The two remaining phases were separated by vacuum filtration. Then, the solid fraction was washed thoroughly with distilled water to remove any residual lactic acid. The obtained dark brown solid fraction (Figure 1) was labeled as raw chitin (RCH) and then stored for further purification. The demineralization solution (DMS) containing calcium (Ca^2+^) and lactate (CH_3_CHOHCOO^−^) ions was retained for further preparation of calcium lactate.

### 3.3. Chitin Purification

The obtained RCH was purified by deproteinization and decolorization. By the removal-protein method, RCH powder was mixed with 1 M sodium hydroxide at a 1:10 (*w*/*v*) ratio, and stirred at 600 rpm for 1 h. Then, the mixture containing the sodium hydroxide solution and a dark black solid of chitin (Figure 1) was separated by draining off the solution, and the deproteinized chitin (DPC) was obtained for further decolorization. Typically, a dark black DPC powder was soaked in 10% *w*/*v* H_2_O_2_ at a 1:10 (*w*/*v*) ratio at 90 °C with a stirring speed of 600 rpm for 0.5 h. After that, the mixture was filtered and washed thoroughly with distilled water to eliminate any remaining H_2_O_2_. The pale brown solid obtained (Figure 1) was labeled as purified chitin (PCH).

### 3.4. Chitosan Derivation

The purified chitin (PCH) was converted to chitosan by deacetylation in a strong base. PCH was suspended in 10 M NaOH at a 1:20 (*w*/*v*) ratio at 100 °C with continuous stirring at 600 rpm for 4 h. Then, the resulting mixture is left to cool down at room temperature. After that, 20 mL of 99% ethanol was added to the resulting mixture and stirred at 600 rpm for 30 min. The presence of alcohol can solidify chitosan by reducing the solvent’s polarity, causing the chitosan molecules to form a solid structure, as reported by Lu et al. [72]. Finally, a pale yellow powder was obtained and isolated by filtration, washed with acetone three times, and dried at 60 °C for 2 h, which is called chitosan (CTS).

### 3.5. Calcium Lactate Production

The demineralization solution (DMS) containing calcium and lactate ions was prepared according to Equation (1) and then dried by simple evaporation to produce calcium lactate powder. A total of 1000 mL of the DMS was dried in an oven at 60 °C for 24 h, yielding a white chunk of calcium lactate. The percentage yield of calcium lactate was calculated using the calcium ion concentration as the limiting reagent, estimated at 0.96 M, based on the purity of calcium in the mussel shell determined by XRF. After that, a white chunk of calcium lactate was ground and sieved through a 100-mesh sieve to obtain a fine white powder (Figure 1) of calcium lactate, labeled as CCL.

### 3.6. Characterization

All prepared RCH, PCH, CTS, and CCL samples were characterized by scientific instruments to verify their chemical and physical properties. Fourier Transform Infrared (FTIR) Spectroscopy was performed by using a PerkinElmer Spectrum GX spectrophotometer (Spectrum GX, PerkinElmer Inc., Waltham, MA, USA) to record the infrared spectra of the samples from the range between 4000 and 400 cm^−1^, with the scan number of 32 scans at a resolution of 4 cm^−1^. Each sample (30 mg) was homogeneously mixed with AR-grade potassium bromide (KBr) powders. The crystalline structures of the samples were determined using X-Ray Diffraction (XRD) with a Rigaku MiniFlex diffractometer (MiniFlex, Rigaku Corporation, Tokyo, Japan). The measurements were carried out with Cu Kα irradiation (40 kV, 32 mA) from scan angles between 5° and 60° at a scan speed of 0.04°/s. Thermal stability and decomposition patterns were evaluated using a PerkinElmer Pyris Diamond thermogravimetric analyzer (Pyris Diamond, PerkinElmer Inc., Waltham, MA, USA). The analysis was performed in α-Al_2_O_3_ crucibles under a nitrogen (N_2_) atmosphere, with temperatures ranging from 30 to 900°C using a constant heating rate of 10°C/min. The morphologies of the prepared samples were observed using a Scanning Electron Microscope (LEO 1530, Carl Zeiss AG, Oberkochen, Germany). Before analysis, all samples were coated with a thin layer of gold using a sputtering technique to enhance conductivity [73].

The degree of deacetylation (DD) was estimated from the FTIR result of CTS according to the method described by Kaewprachu and Jaisan [30]. The absorbance at A1650 and A3450 cm^−1^ indicate absolute heights of absorption bands of amide and hydroxyl groups, respectively. The DD (%) was calculated using Equation (6):

(6) $$DD=100\times [(\frac{A1650}{A3450})\times \frac{100}{1.33}]$$

The molecular weight (Mw) of the chitosan was further determined from the intrinsic viscosity (η) of the chitosan solution in 0.3 M acetic acid/0.2 M sodium acetate solution, using an Ostwald capillary-type viscometer [45], and then calculated according to the Mark–Houwink relationship (Equation (7)):

[η] = KM^−α^ (7)

where K and α are empirical constants specific to a given polymer–solvent system under defined temperature conditions.

## 4. Conclusions

Mussel shell biowaste was simultaneously converted into chitin, chitosan, and calcium lactate using a green bio-upcycling route with lactic acid as the demineralizing agent. From 100 g of mussel shells, 2.37 g raw chitin and 2.15 g purified chitin were obtained, while direct evaporation of the demineralization solution yielded 275.87 g calcium lactate pentahydrate. Multi-technique characterization consistently verified the properties of all products obtained. FTIR showed α-chitin amide I/II/III bands with the expected N–H/O–H groups, while characteristic peaks of the acetyl group (-COCH_3_) decreased after deacetylation in chitosan. For calcium lactate, the characteristic peaks are similar to those synthesized by other calcium sources. XRD displayed the broad chitin reflections at 2θ around 9–10° and 19–20°, chitosan peaks near 18°, 38°, and 44°, and calcium lactate patterns matching standard JCPDS data of Ca(CH_3_CHOHCOO)_2_·5H_2_O. TG/DTG evidenced water loss and polymer depolymerization/cracking for chitin/chitosan. For calcium lactate, the TG/DTG graph shows dehydration (~5 H_2_O), followed by conversion to CaCO_3_ and decarbonation to CaO. SEM corroborated purification-driven surface smoothing of chitin, sheet-like chitosan domains, and well-formed timber-like calcium lactate microcrystals. Importantly, the calcium lactate showed higher purity (XRF: 98.3% CaO-equivalent) than reported previously. Across FTIR, XRD, and TGA, the properties of chitin and chitosan closely match those of products derived from shrimp and crab shells in previous research. Overall, this first simultaneous production of chitin, chitosan, and calcium lactate from mussel shells provides a value-added pathway for sustainable waste management and advances the Bio-Circular-Green economy.

## Data Availability

All data are fully available without restriction.

## References

[1] Liu T.G., Li B., Huang W., Lv B., Chen J., Zhang J.X., Zhu L.P. (2009). Effects and kinetics of a novel temperature cycling treatment on the N-deacetylation of chitin in alkaline solution. Carbohydr. Polym..

[2] Özel N., Elibol M. (2021). A review on the potential uses of deep eutectic solvents in chitin and chitosan related processes. Carbohydr. Polym..

[3] Summa D., Lanzoni M., Castaldelli G., Fano E.A., Tamburini E. (2022). Trends and Opportunities of Bivalve Shells’ Waste Valorization in a Prospect of Circular Blue Bioeconomy. Resources.

[4] Fishery Statistics Group, Department of Fisheries Ministry of Agriculture and Cooperatives (2022). Statistics of Marine Shellfish CULTURE Survey. https://www4.fisheries.go.th/local/file_document/20250718085808_new.pdf.

[5] Sathiyavimal S., Vasantharaj S., Mattheos N., Pugazhendhi A., Subbalekha K. (2024). Mussel shell-derived biogenic hydroxyapatite as reinforcement on chitosan-loaded gentamicin composite for antibacterial activity and bone regeneration. Int. J. Biol. Macromol..

[6] Medina Uzcátegui L.U., Vergara K., Martínez Bordes G. (2022). Sustainable alternatives for by-products derived from industrial mussel processing: A critical review. Waste Manag. Res..

[7] Shon B.-H., Jung J.-H., Lee J.-J., Yoo J.-S.Y., Lee G.-W., Achilias D.S. (2012). Reuse of Waste Shells as a SO_2_/NO_x_ Removal Sorbent. Material Recycling-Trends and Perspectives.

[8] Laohavisuti N., Boonchom B., Boonmee W., Chaiseeda K., Seesanong S. (2021). Simple recycling of biowaste eggshells to various calcium phosphates for specific industries. Sci. Rep..

[9] Mititelu M., Stanciu G., Drăgănescu D., Ioniță A.C., Neacșu S.M., Dinu M., Stefan-van Staden R.I., Moroșan E. (2021). Mussel Shells, a Valuable Calcium Resource for the Pharmaceutical Industry. Mar. Drugs.

[10] Seesanong S., Laosinwattana C., Boonchom B. (2019). A simple rapid route to synthesize monocalcium phosphate monohydrate using calcium carbonate with different phases derived from green mussel shells. J. Mater. Environ. Sci..

[11] Isnaini M.D., Jongsomjit B., Yip A.C.K., Phisalaphong M. (2025). Waste-derived CaO from green mussel shells as a highly stabilized and superior sorbent for cyclic CO_2_ capture. Clean. Eng. Technol..

[12] Ismail R., Cionita T., Shing W.L., Fitriyana D.F., Siregar J.P., Bayuseno A.P., Nugraha F.W., Muhamadin R.C., Junid R., Endot N.A. (2022). Synthesis and Characterization of Calcium Carbonate Obtained from Green Mussel and Crab Shells as a Biomaterials Candidate. Materials.

[13] Dugaich A.P.C., Barboza A.d.S., Silva M.G.e., Nörnberg A.B., Maraschin M., Badaró M.M., Silva D.F.d., Campos C.E.M.d., Santinoni C.d.S., Stolf S.C. (2026). Sustainable Valorization of Mussel Shell Waste: Processing for Calcium Carbonate Recovery and Hydroxyapatite Production. J. Funct. Biomater..

[14] Murphy J.N., Morgan M.A., Christian-Robinson S., Fitzgerald M.M., Kerton F.M. (2025). Optimized process to produce calcium acetate from waste blue mussel shells and its use as a de-icer. Can. J. Chem..

[15] Mititelu M., Moroșan E., Nicoară A.C., Secăreanu A.A., Musuc A.M., Atkinson I., Pandele Cusu J., Nițulescu G.M., Ozon E.A., Sarbu I. (2022). Development of Immediate Release Tablets Containing Calcium Lactate Synthetized from Black Sea Mussel Shells. Mar. Drugs.

[16] Punthipayanon S., Chanwetprasat P., Seesanong S., Boonchom B., Rungrojchaipon P., Laohavisuti N., Boonmee W. (2025). Influence of Organic Solvent on the Physicochemical Characteristics of Calcium Citrate Prepared from Mussel Shell Waste. Processes.

[17] Souidi A., Maaloufa Y., Amazal M., Atigui M., Oubeddou S., Mounir S., Idoum A., Aharoune A. (2024). The effect of mussel shell powder on the thermal and mechanical properties of plaster. Constr. Build. Mater..

[18] Azarian M.H., Sutapun W. (2022). Biogenic calcium carbonate derived from waste shells for advanced material applications: A review. Front. Mater..

[19] De Pascale B., Tarsi G., Tataranni P., Sangiorgi C. (2024). Potential application of waste bivalve shells as recycled filler in porous asphalt concrete through rheo-mechanical analysis. Resour. Conserv. Recycl..

[20] Cadano J.R., Jose M., Lubi A.G., Maling J.N., Moraga J.S., Shi Q.Y., Vegafria H.M., VinceCruz-Abeledo C.C. (2021). A comparative study on the raw chitin and chitosan yields of common bio-waste from Philippine seafood. Environ. Sci. Pollut. Res..

[21] Pratama G., Munandar A., Surilayani D., Rizky J.A., Hasanah A.N., Haryati S., Meata B.A., Nuryadin D.F.E., Aditia R.P. (2023). Characteristics of crab shells and green mussel shells as potential chitosan material from Karangantu, Banten, Indonesia. Proceedings of the IOP Conference Series: Earth and Environmental Science.

[22] Rinaudo M. (2006). Chitin and chitosan: Properties and applications. Prog. Polym. Sci..

[23] Bai L., Liu L., Esquivel M., Tardy B.L., Huan S., Niu X., Liu S., Yang G., Fan Y., Rojas O.J. (2022). Nanochitin: Chemistry, Structure, Assembly, and Applications. Chem. Rev..

[24] Fernández-Marín R., Morales A., Erdocia X., Iturrondobeitia M., Labidi J., Lizundia E. (2024). Chitosan–Chitin Nanocrystal Films from Lobster and Spider Crab: Properties and Environmental Sustainability. ACS Sustain. Chem. Eng..

[25] Pillai C.K.S., Paul W., Sharma C.P. (2009). Chitin and chitosan polymers: Chemistry, solubility and fiber formation. Prog. Polym. Sci..

[26] Salaün F., Chen Y., Ferri A., Giraud S., Roy J.C., Jinping G., Chen G. (2017). Solubility of Chitin: Solvents, Solution Behaviors and Their Related Mechanisms. Solubility of Polysaccharides.

[27] Ravi Kumar M.N.V. (2000). A review of chitin and chitosan applications. React. Funct. Polym..

[28] Dutta P.K., Dutta J., Tripathi V.S. (2004). Chitin and chitosan: Chemistry, properties and applications. J. Sci. Ind. Res..

[29] Al Shaqsi N.H.K., Al Hoqani H.A.S., Hossain M.A., Al Sibani M.A. (2020). Optimization of the demineralization process for the extraction of chitin from Omani *Portunidae segnis*. Biochem. Biophys. Rep..

[30] Kaewprachu P., Jaisan C. (2023). Physicochemical Properties of Chitosan from Green Mussel Shells (*Perna viridis*): A Comparative Study. Polymers.

[31] Seesanong S., Seangarun C., Boonchom B., Phutphat S., Rungrojchaipon P., Montri N., Thompho S., Boonmee W., Laohavisuti N. (2023). Efficient, Green, and Low-Cost Conversion of Bivalve-Shell Wastes to Value-Added Calcium Lactate. ACS Omega.

[32] Seangarun C., Seesanong S., Boonchom B., Laohavisuti N., Rungrojchaipon P., Boonmee W., Punthipayanon S., Thongkam M. (2025). Extraction of Chitin, Chitosan, and Calcium Acetate from Mussel Shells for Sustainable Waste Management. Int. J. Mol. Sci..

[33] Sharma A., Singh S., Khare S.K., Sharma A., Tiwari R., Nain L. (2022). 11-Green lactic acid production using low-cost renewable sources and potential applications. Production of Top 12 Biochemicals Selected by USDOE from Renewable Resources.

[34] Bisht M., Macário I.P.E., Neves M.C., Pereira J.L., Pandey S., Rogers R.D., Coutinho J.A.P., Ventura S.P.M. (2021). Enhanced Dissolution of Chitin Using Acidic Deep Eutectic Solvents: A Sustainable and Simple Approach to Extract Chitin from Crayfish shell Wastes as Alternative Feedstocks. ACS Sustain. Chem. Eng..

[35] Saravana P.S., Ho T.C., Chae S.-J., Cho Y.-J., Park J.-S., Lee H.-J., Chun B.-S. (2018). Deep eutectic solvent-based extraction and fabrication of chitin films from crustacean waste. Carbohydr. Polym..

[36] McReynolds C., Adrien A., Petitpas A., Rubatat L., Fernandes S.C.M. (2022). Double Valorization for a Discard—α-Chitin and Calcium Lactate Production from the Crab *Polybius henslowii* Using a Deep Eutectic Solvent Approach. Mar. Drugs.

[37] Daengprok W., Garnjanagoonchorn W., Mine Y. (2002). Fermented pork sausage fortified with commercial or hen eggshell calcium lactate. Meat Sci..

[38] Linares-Morales J.R., Gutiérrez-Méndez N., Rivera-Chavira B.E., Pérez-Vega S.B., Nevárez-Moorillón G.V. (2018). Biocontrol Processes in Fruits and Fresh Produce, the Use of Lactic Acid Bacteria as a Sustainable Option. Front. Sustain. Food Syst..

[39] Devesa-Rey R., Fernández N., Cruz J.M., Moldes A.B. (2011). Optimization of the dose of calcium lactate as a new coagulant for the coagulation–flocculation of suspended particles in water. Desalination.

[40] Vijay K., Murmu M. (2019). Effect of calcium lactate on compressive strength and self-healing of cracks in microbial concrete. Front. Struct. Civ. Eng..

[41] Nair P.A., Ramesh P. (2012). Synthesis and characterization of calcium-containing polyurethane using calcium lactate as a chain extender. Polym. J..

[42] Research C. (2024). Calcium Lactate Market: Growth, Trends, COVID-19 Impact, and Forecasts (2024–2032). https://www.credenceresearch.com/report/calcium-lactate-market.

[43] Younes I., Rinaudo M. (2015). Chitin and Chitosan Preparation from Marine Sources. Structure, Properties and Applications. Mar. Drugs.

[44] Suneeta K., Rath P., Annamareddy S. (2016). Chitosan from shrimp shell (*Crangon crangon*) and fish scales (*Labeorohita*): Extraction and characterization. Afr. J. Biotechnol..

[45] Triunfo M., Tafi E., Guarnieri A., Salvia R., Scieuzo C., Hahn T., Zibek S., Gagliardini A., Panariello L., Coltelli M.B. (2022). Characterization of chitin and chitosan derived from *Hermetia illucens*, a further step in a circular economy process. Sci. Rep..

[46] Duan C., Meng X., Meng J., Khan M.I.H., Dai L., Khan A., An X., Zhang J., Huq T., Ni Y. (2019). Chitosan as A Preservative for Fruits and Vegetables: A Review on Chemistry and Antimicrobial Properties. J. Bioresour. Bioprod..

[47] Lu J.Y., Carter E., Chung R.A. (1980). Use of Calcium Salts for Soybean Curd Preparation. J. Food Sci..

[48] Torres S., Boetzel R., Gatimu E., Gomes D.Z., King F., Kocks G., Jones R., Day C., Lewen N., Harris L. (2022). ICH Q3D Drug Product Elemental Risk Assessment: The Use of An Elemental Impurities Excipients Database. J. Pharm. Sci..

[49] Xiong A., Ruan L., Ye K., Huang Z., Yu C. (2023). Extraction of Chitin from Black Soldier Fly (*Hermetia illucens*) and Its Puparium by Using Biological Treatment. Life.

[50] Focher B., Naggi A., Torri G., Cosani A., Terbojevich M. (1992). Chitosans from *Euphausia superba*. 2: Characterization of solid state structure. Carbohydr. Polym..

[51] Narasagoudr S.S., Hegde V.G., Chougale R.B., Masti S.P., Dixit S. (2020). Influence of boswellic acid on multifunctional properties of chitosan/poly (vinyl alcohol) films for active food packaging. Int. J. Biol. Macromol..

[52] Bhavsar P.S., Dalla Fontana G., Zoccola M. (2021). Sustainable Superheated Water Hydrolysis of Black Soldier Fly Exuviae for Chitin Extraction and Use of the Obtained Chitosan in the Textile Field. ACS Omega.

[53] Song C., Yu H., Zhang M., Yang Y., Zhang G. (2013). Physicochemical properties and antioxidant activity of chitosan from the blowfly *Chrysomya megacephala* larvae. Int. J. Biol. Macromol..

[54] Dechapinan S., Judprasong K., On-nom N., Tangsuphoom N. (2017). Calcium from Pacific White Shrimp (*Litopeneaus vannamei*) Shells: Properties and Function as Fortificant in Soy Milk. Food Appl. Biosci. J..

[55] Yen M.-T., Mau J.-L. (2007). Physico-chemical characterization of fungal chitosan from shiitake stipes. LWT-Food Sci. Technol..

[56] Tansman G., Kindstedt P., Hughes J. (2014). Powder x-ray diffraction can differentiate between enantiomeric variants of calcium lactate pentahydrate crystal in cheese. J. Dairy Sci..

[57] Paulino A.T., Simionato J.I., Garcia J.C., Nozaki J. (2006). Characterization of chitosan and chitin produced from silkworm crysalides. Carbohydr. Polym..

[58] Stawski D., Rabiej S., Herczyńska L., Draczyński Z. (2008). Thermogravimetric analysis of chitins of different origin. J. Therm. Anal. Calorim..

[59] Fernández-Marín R., Hernández-Ramos F., Salaberria A.M., Andrés M.Á., Labidi J., Fernandes S.C.M. (2021). Eco-friendly isolation and characterization of nanochitin from different origins by microwave irradiation: Optimization using response surface methodology. Int. J. Biol. Macromol..

[60] Sajomsang W., Gonil P. (2010). Preparation and characterization of α-chitin from cicada sloughs. Mater. Sci. Eng. C.

[61] Kaya M., Baran T., Karaarslan M. (2015). A new method for fast chitin extraction from shells of crab, crayfish and shrimp. Nat. Prod. Res..

[62] Barbosa H.F.G., Francisco D.S., Ferreira A.P.G., Cavalheiro É.T.G. (2019). A new look towards the thermal decomposition of chitins and chitosans with different degrees of deacetylation by coupled TG-FTIR. Carbohydr. Polym..

[63] Nieto J.M., Peniche-Covas C., Padro´n G. (1991). Characterization of chitosan by pyrolysis-mass spectrometry, thermal analysis and differential scanning calorimetry. Thermochim. Acta.

[64] Qu X., Wirsén A., Albertsson A.C. (2000). Effect of lactic/glycolic acid side chains on the thermal degradation kinetics of chitosan derivatives. Polymer.

[65] Cheong S.H. (2016). Physicochemical properties of calcium lactate prepared by single-phase aragonite precipitated calcium carbonate. Research. J. Pharm. Biol. Chem. Sci..

[66] Sakata Y., Shiraishi S., Otsuka M. (2005). Characterization of dehydration and hydration behavior of calcium lactate pentahydrate and its anhydrate. Colloids Surf. B Biointerfaces.

[67] Sronsri C., Boonchom B. (2017). Deconvolution technique for the kinetic analysis of a complex reaction and the related thermodynamic functions of the formation of LiMn_0.90_Co_0.05_Mg_0.05_PO_4_. Chem. Phys. Lett..

[68] Sronsri C., Boonchom B. (2018). Thermal kinetic analysis of a complex process from a solid-state reaction by deconvolution procedure from a new calculation method and related thermodynamic functions of Mn_0.90_Co_0.05_Mg_0.05_HPO_4_·3H_2_O. Trans. Nonferrous Met. Soc. China.

[69] Criado Y.A., Arias B., Abanades J.C. (2018). Effect of the Carbonation Temperature on the CO_2_ Carrying Capacity of CaO. Ind. Eng. Chem. Res..

[70] Mohan K., Muralisankar T., Jayakumar R., Rajeevgandhi C. (2021). A study on structural comparisons of α-chitin extracted from marine crustacean shell waste. Carbohydr. Polym. Technol. Appl..

[71] de Queiroz Antonino R., Lia Fook B.R.P., de Oliveira Lima V.A., de Farias Rached R., Lima E.P.N., da Silva Lima R.J., Peniche Covas C.A., Lia Fook M.V. (2017). Preparation and Characterization of Chitosan Obtained from Shells of Shrimp (*Litopenaeus vannamei Boone*). Mar. Drugs.

[72] Lu H., Wang W., Wang A. (2015). Ethanol–NaOH solidification method to intensify chitosan/poly(vinyl alcohol)/attapulgite composite film. RSC Adv..

[73] Sronsri C., Noisong P., Danvirutai C. (2014). Synthesis, non-isothermal kinetic and thermodynamic studies of the formation of LiMnPO_4_ from NH_4_MnPO_4_·H_2_O precursor. Solid State Sci..

